# Supplementary material for: Are carbon emissions trading and green financial instruments synergistic? -Comprehensive quantitative research based on content analysis
Source: PLoS One. 2024 Mar 7;19(3):e0298601. doi: 10.1371/journal.pone.0298601 (PMC10919601; doi:10.1371/journal.pone.0298601)
Supplement: S1 Data — (ZIP) [file pone.0298601.s001.zip › Data/地级市Z_F工作报告.zip!/║■▒▒/╢⌡╓▌╩╨/└┤╫╘╣1⁄2╓┌║┼í╛┬φ┐╦╩2╛▌═°í┐_2008.docx]

各位代表：

　　现在，我代表第五届市人民政府，向市第六届人民代表大会第一次会议报告过去四年的工作，对今后四年及今年的工作提出建议，请予审议，并请市政协委员和列席人员提出意见。

　　一、过去四年工作的回顾

　　过去四年，是鄂州建市以来发展最好最快、人民群众受益最多的时期之一。四年来，在省委、省政府和市委的正确领导下，本届政府带领全市人民认真贯彻落实科学发展观，抢抓促进中部崛起和建设武汉城市圈的重大机遇，团结拼搏、改革创新、扩大开放，经济发展呈现出速度加快、结构优化、效益提高和后劲增强的良好态势，社会事业全面进步，人民生活显著改善。市五届人大一次会议确定的主要预期目标全面超额完成。

　　——经济发展提速。加快融入武汉城市圈，加速推进新型工业化，加大“对接武汉、深耕广东、开发江浙”力度，经济进入高速发展期。2004年—2007年，地区生产总值年均增长13.2%，增幅是建市以来最快的时期之一；2007年达到208.7亿元，同比增长15.8%，增幅居全省第三位。财政总收入年均增长24.5%，是建市以来增长最快的时期，2007年达到20.95亿元，同比增长26.2%；地方一般预算收入达到8.5亿元，同比增长31.6%，增幅居全省第一位。四年累计完成全社会固定资产投资276.5亿元，年均增长25.5%，2007年达到105亿元，同比增长45.7%，增幅居全省第一位。

　　——结构效益优化。全面推进经济结构战略性调整，培育壮大市场主体，支持区级经济发展，狠抓节能减排，经济结构日益优化，发展质量明显提高。四年新增规模以上工业企业100家，2007年达到302家，工业企业经济效益综合指数比2003年提高56.9个百分点。列入两改计划的131家国有企业基本完成改制任务。市场主体日益多元，全市民营企业达到3361户，个体工商户达到15223家。旅游、房地产等服务业加速发展，2007年第三产业实现增加值68.4亿元，年均增长9.6%。2007年实现高新技术产业增加值19.76亿元，年均增长22.1%。国地两税收入占地区生产总值的比重达到9.2%，比2003年提高2.7个百分点。外贸出口（海关数）年均增长19.3%，2007年突破1亿美元，比2003年翻一番。“多佳”商标获中国驰名商标，“顾地牌”管材管件获“中国名牌”。“武昌鱼”原产地证明商标在国家工商总局成功注册。8个农产品获得国家绿色食品标识，29个农产品获得省级无公害产地、无公害产品认证。胡柚基地成为省级绿色食品农业标准化示范区。

　　——发展后劲增强。重大基础设施项目相继实施，高新大道、红莲大道、庙岭立交、316国道改造等工程建成通车，汉鄂快速通道完成前期工作，葛湖路、樊口大桥等重点工程正在抓紧建设。开发区建设取得重大突破，投入15亿元新建鄂州开发区、提升葛华科技新城和花湖工贸新城，产业承载力和要素吸纳力显著增强。四年累计引进5000万元以上项目313个，开工项目133个；新上亿元项目65个，是1983年—2003年的4倍。　招商引资总额年均增长35.8%，2007年达到5.7亿美元，是2003年的3.4倍。鄂钢冷轧薄板、宽厚板，武钢球团厂一期，鄂州电厂二期，世纪新峰，比克电池，宝业产业化等一批重大项目相继启动或建成投产。四年共批回建设用地1.65万亩，其中近两年批回1.38万亩，用地紧张局面得以缓解。金融运行质量提高，获得“A级信用城市”称号，荣膺“中国优秀旅游城市”、“国家级生态示范区”和“中部最佳投资城市”称号。

　　——城乡变化明显。“一主三新十特”的城镇体系基本形成。全面开展“四城联创”，城市发展呈现新的格局。四年来共完成城市建设投资21.6亿元，完成交通建设投资15.1亿元，均是建市以来投入最多的时期。　新增建成区面积8平方公里。公路密度居全省第1位。整合成立了新的城投公司。完成了江碧大道、沿江大道、滨湖路等干道建设，启动了寿昌大道、吴都大道、江滩整治、洋澜湖截污、城区污水处理厂、百洪垃圾处理场等工程。鄂州火车站升级为二级站，华容火车站竣工。取缔主城区正三轮摩托载客运营。鞭炮禁改限、集贸市场集中整治和环保“七个专项”整顿初见成效。梁子湖水生植被修复保护模式在全国推广。全面取消农业税，乡镇综合配套改革进展顺利，初步建立了“以钱养事”新机制。大力培育文明乡风，农村精神文明建设得到加强。在全省率先进行城乡一体化改革试点。投入1.3亿元，在全省率先实行城乡供水一体化，让农村33.2万人喝上了自来水。在全省率先实现了村村通水泥公路，率先实现农村渡口全部达标。投入7800万元，维修改造200座抗旱排涝泵站。投入1.87亿元，整理土地9.89万亩。投入1730万元，对30个重点村实施整村推进扶贫开发。发放农机补贴204万元，新增农机具2.08万台（套）。大力实施“阳光工程”和“雨露计划”，培训转移农村劳动力3.87万人。

　　——人民生活改善。全市城镇居民人均可支配收入年均增长11.9%,2007年达到10827元，超过全省平均增幅2.9个百分点。农民人均纯收入年均增长11.6%，2007年达到4393元，超过全省平均水平。落实国家惠农资金3806万元。发放小额担保贷款1490万元，新增就业岗位8.6万个。在全省地级市中率先启动农村低保，全市2.4万名城镇低保人员和1.2万名国有困难企业退休职工全部参加医疗保险。发放大病救助资金1170万元，缓解了困难群众看病难。整体纳入全国新型农村合作医疗试点，到2007年末共有57.43万人参保，参合率达84.9%。投入3000万元，完成了市传染病医院、14个国家农村卫生服务体系等项目建设。投入资金2000万元，新建和改扩建福利院23所。提供住房公积金贷款3.33亿元，使6215户家庭圆了住房梦。筹资460万元，解决了505户农村低保无房户和危房户的住房问题。筹资150万元，对城镇1500户住房困难的最低收入家庭实施了租赁住房补贴和租金核减。筹资2973万元，对40737名贫困生免费提供教科书，全部免除了农村义务教育阶段学生学杂费，对2197名贫困家庭寄宿生提供生活补助。四年累计投入办实事资金4亿多元，受益人口达30多万人。

　　——社会和谐稳定。制定和完善了《市人民政府议事和决策规程》等一系列规范性文件，加大了行政执法行为的监督力度，行政执法质量明显提高。积极推动政务公开，创办了《鄂州市人民政府公报》，建立了市政府与市人大市政协联系会议制度、市政府新闻发言人制度和市长在线访谈制度。开通了“亲民热线”、“市长信箱”。四年共办理人大代表议案建议349件，政协委员提案799件，回复率达100%，满意率达98%。　开展发展环境万人评活动，市直部门服务意识进一步增强。对行政审批事项进行全面清理，全市行政审批事项由1005项减至661项。认真开展中介机构与行政事业单位脱钩和整顿规范工作。部门预算、政府采购、非税收入管理和政府收支分类改革成效明显。审计工作进一步加强，四年来审计项目423个，查处各类违纪违规金额5.13亿元。加强社会治安综合治理，深入开展法制教育“六进”活动，刑事发案总量呈下降趋势。出生人口性别比逐年下降，人口自然增长率控制在5‰以内。市场监管力度加大，治理商业贿赂、打击传销、产品质量和食品安全专项治理工作取得明显成效，安全生产保持平稳态势，信访工作得到加强。圆满完成了“八艺节”和“六城会”承办任务。开展“周周乐”广场文化活动，实施农民体育健身和城乡电影“2131”工程，丰富了群众的文体生活。

　　与此同时，统计、物价、粮食、烟草、广播电视、民族宗教、外事侨务、对台、档案方志、工会、青年、妇女、儿童、残联、供电、保险、邮政、通讯、石油石化、新闻出版、人防、老龄、气象和国防教育、民兵建设等工作都取得了新的成绩。

　　各位代表，鄂州经济和社会发展取得的成绩来之不易，这是市委正确决策和坚强领导的结果，是市人大、市政协和全市各民主党派、工商联及无党派人士关心和支持的结果，是全市人民团结奋斗的结果，也得益于历届市委市政府打下的良好基础。借此机会，我谨代表市人民政府向辛勤工作在各个岗位上的广大干部群众，向各位人大代表、政协委员，向各民主党派、工商联、无党派人士、人民团体和社会各界人士，向为鄂州发展作出贡献的各位老同志，向驻军指战员、武警官兵以及所有来鄂州的投资者、劳动者和海内外朋友表示崇高的敬意和衷心的感谢！

　　过去四年，我们在加快发展的实践中积累了一些宝贵经验，值得在今后的工作中借鉴和发扬。

　　一是必须注重解放思想，从战略全局谋划发展。近年来鄂州的快速发展，主要得益于坚持解放思想，从实际出发谋划发展，以全局意识统筹发展，用科学视野推动发展。为了提升发展层次，突破行政区划制约，跳出鄂州看鄂州，立足武汉城市圈和鄂东城市群对鄂州发展进行了重新定位；为了拓展发展空间，根据经济流向和发展潜力，对城镇布局进行了重新规划；为了增加发展后劲和动力，强力推进招商引资和项目建设，建设和提升了“两区三城”。实践证明，只有始终注重对经济社会发展的全局性、前瞻性、战略性问题进行深入研究、不懈探索，才能真正实现又好又快发展。

　　二是必须注重改革开放，以开放促改革促发展。鄂州的希望在改革开放，潜力也在改革开放。改革开放是富民之基、强市之路。只有不断深化改革开放，才能促进思想观念的转变，建立起与科学发展观和社会主义市场经济相适应的体制机制；才能更好地承接产业转移和大城市的辐射带动，吸引更多优势资源和优秀人才聚集鄂州、建设鄂州，激发创业活力，壮大市场主体。

　　三是必须注重“打基础、管长远”，不断增强发展后劲。当前工作是长远发展的基础,长远发展是当前工作的远景。既要志存高远谋划宏伟目标，又要求真务实打好坚实基础。特别是事关长远的发展规划、事关全局的重大项目、事关民生的惠民工程，要舍得花投入、舍得花精力，即使短时间内难见成效，但只要对长远有利、对全局有利、对市民有利，就要义无反顾地去推动、去落实。

　　四是必须注重制度建设，依法规范行政行为。发展提速需要优化环境，优化环境需要规范行政行为，规范行政行为要靠制度建设。近几年市政府加快完善行政决策机制、行政运行机制、行政监督机制和行政问责机制，对建设法治政府、效能政府和廉洁政府产生了积极作用。更加注重制度建设，逐步建立起科学行政、民主行政、依法行政体系，建立起与时代需要和人民愿望相契合的制度和机制。

　　五是必须注重民生改善，努力促进社会和谐稳定。改善民生是加快发展的目的和归宿，和谐稳定是加快发展的基础和前提。要把改善民生作为正确处理改革发展稳定关系的结合点，让人民群众从改革发展中真正得到实惠。要进一步加强社会治安综合治理，增强人民群众安全感；要进一步拓宽社情民意表达渠道，积极预防和妥善处置社会矛盾，维护群众利益和社会稳定。

　　总结回顾过去的四年，我们也清醒地认识到，本届政府的工作在某些方面与全市人民的期待还有一定差距，经济社会发展中仍存在一些突出矛盾和困难，主要是：经济规模仍然偏小，区乡财力比较薄弱，结构调整任务较重，环境保护压力较大；要素制约问题比较突出，新经济领域的专业人才缺乏、土地供应紧张、中小企业融资难等问题不同程度地影响了发展；城乡居民收入差距较大，农民收入稳定增长的渠道还不宽，失地农民生活保障机制有待建立和完善；政府自身改革和建设有待进一步加强，一些部门办事效率不高，少数政府工作人员服务意识和依法行政观念不强、作风不实。解决这些矛盾和问题，是政府今后的艰巨任务，我们将认真面对，统筹谋划，切实加以解决。

　　二、今后四年工作的建议

　　今后四年，是鄂州发展充满希望、人民满怀期盼的四年，是经济实现跨越、社会和谐发展的四年，是融入武汉城市圈、探索新的发展模式的四年，是建设宜居宜业大城市、群众生活走向富裕的四年。新的四年，我们面临不可多得的战略机遇：国家批准武汉城市圈为资源节约型和环境友好型社会建设综合配套改革试验区，省委省政府批复鄂州为城乡一体化试点城市，为我们深化改革、创新体制机制提供了新的平台；国际资本加速向中国转移，沿海产业加速向中西部转移，为我们扩大开放、招商引资提供了新的契机；全市上下励精图治，经济发展的内生动力不断增强，一批亿元以上重大项目正在实施，为经济跨越、社会和谐打下了坚实基础。在确保节能环保、质量效益的前提下，能发展多快就发展多快，既是全市人民的迫切愿望，也是省委、省政府对鄂州的殷切希望，更是新一届政府必须牢牢把握的第一要务。我们要进一步增强责任感和紧迫感，找准发展定位，抢抓发展机遇，切实把区位优势转化为经济优势，把政策优势转化为发展优势，奋力实现经济社会又好又快发展。

　　今后四年政府工作的总体要求是：认真贯彻落实党的十七大、省第九次党代会、省十一届人大一次会议和市五次党代会精神，以科学发展观统领全局，紧紧抓住武汉城市圈“资源节约型、环境友好型”综合配套改革试验区建设的机遇，率先融入武汉城市圈，率先实现城乡一体化，努力建设“两型社会”先行区，为把鄂州建设成为适宜人居、适宜创业的大城市打下坚实基础。按照市委五届三次会议确立的目标，到2011年，力争在2007年的基础上人均GDP翻一番，人均财政收入翻一番。力争四年累计固定资产投资在前四年投资总额的基础上翻两番，达到1000亿元左右；工业销售收入在2007年的基础上实现翻两番，达到1000亿元左右；累计新增建成区面积32平方公里左右，比前四年增量总和翻两番；累计新增城镇人口16万人左右，比前四年新增人口翻两番。群众普遍关心的就业、看病、上学、社会保障、农村安全饮水、城乡居民住房、节能减排等问题明显缓解或基本解决，全市经济更加发达、法制更加完善、文化更加繁荣、社会更加和谐、环境更加优美、人民更加富裕。根据上述总体要求和奋斗目标，本届市政府经过认真研究，对今后四年的政府工作提出以下建议。

　　（一）围绕率先融入武汉城市圈，努力推进跨越发展

　　紧紧把握武汉城市圈被国家确定为“两型社会”建设综合配套改革试验区的重大机遇，加快推进与武汉、黄石等周边城市的规划同筹、交通同网、产业同链、市场同体、生态同建，率先融入、率先发展。

　　1、以规划为先导，推动基础设施对接。编制完成融入武汉的武鄂一体化对接规划，推进葛华科技新城、红莲湖旅游新城与武汉科技新城、武汉北湖化工新城融合发展，推进花湖工贸新城与黄石主城区融合发展。　尽快开工建设汉鄂快速通道，争取2011年竣工通车；力争开通鄂州与武汉公交，规划筹建武汉至鄂州的轻轨交通干线，形成对接武汉的快速通畅的城际交通网络。依托武汉航运中心优势，加强与武汉的港口合作，提升白浒山、黄柏山、五丈港的吞吐能力，形成功能布局合理、专业分工明确的港口群。加强与黄石路网的连接，全力支持鄂东长江大桥及大广高速公路建设。加快鄂州500千伏变电站建设，并使之与武汉500千伏变电站联网。争取鄂州与武汉电话区号统一，实现同城同网同价。

　　2、以产业为依托，推动经济全面融合。以鄂州开发区为平台，依托武钢鄂钢公司建设钢铁深加工基地。　以葛华科技新城为依托，重点发展电子信息、生物医药、精细化工、装备制造、港口物流业，建设东湖高新区和武汉北湖化工新城的产业配套合作基地。以“一都两湖”为载体，彰显人文生态优势，重点发展运动健身、商务会展、休闲旅游、高端居住、科技研发等产业，建设面向华中、服务武汉的商务运动休闲基地、旅游目的地。以花湖工贸新城为载体，重点发展机械加工、生产性物流、商贸、住宅等产业，建设面向黄石、服务鄂东的工贸基地。

　　3、以市场为纽带，推动优势资源共享。利用同城效应，发挥紧邻东湖智力密集区的优势，深入开展与武汉高校院所的全面合作，使鄂州成为武汉的科技成果转化基地。利用武汉的人才资源，通过柔性引才、优惠政策引才等手段，引进一批鄂州经济社会发展急需的专业技术人才。利用武汉的金融资源，引进各类金融机构在鄂州设立服务网点，增加服务品种，改善企业融资环境。利用武汉、黄石的流通平台和窗口，积极发展产业基地物流、都市配送物流和专业批发市场，形成武汉城市圈重要的物流聚集区。利用鄂州绿色蔬菜、无公害水产等优质农产品资源，把鄂州建成供应武汉及鄂东城市群的农产品集散地。

　　（二）围绕率先实现城乡一体化，努力推进统筹发展

　　紧紧把握省委省政府确定鄂州为全省城乡一体化试点城市的机遇，以城乡规划、产业布局、基础设施、社会保障、公共管理、市场体系六个一体化为切入点，扎实推进新农村建设，努力形成以工补农、以城带乡、城乡共同发展的新格局。

　　1、建立城乡一体的城镇空间布局体系。根据鄂州区位特点和经济流向，加快建设以主城区为中心，以葛华科技新城、红莲湖旅游新城、花湖工贸新城为支撑，以汀祖、燕矶、碧石、长港、段店、蒲团、太和、涂家垴、沼山、梁子十个特色镇为节点的“一主三新十特”城镇空间体系。在全市范围内统一规划布局重大产业发展、公共事业和社会发展项目，实现城乡土地利用、人口分布、产业发展、基础设施、社会事业、生态环境保护等各类专项规划配套对接。加强对市域总体规划、镇村规划、土地利用总体规划等各类规划的统一管理，强化城乡规划的执行和监督；完善城乡规划管理办法，健全市、区、镇（乡）三级规划执法监督管理体制机制，将城镇规划的编制经费纳入同级财政预算；依托乡镇设立新农村、新社区建设管理机构，组织和引导农民有序向城镇和中心村集聚。

　　2、努力实现重点领域的先行突破。进一步放宽户口迁移落户政策，最大限度地降低门槛，取消对农村劳动力进城落户的限制，对流动人口实行居住证管理。建立城乡统筹的就业服务体系，使劳动者依法享受平等就业和自主择业的权利。加强农村富余劳动力转移就业培训，建立政府统一购买培训成果的培训机制。加快建立覆盖城乡的养老、医疗保险制度。缩小城乡最低生活保障差距，逐步实现农村低保应保尽保。进一步提高农村新型合作医疗参保率。逐步建立被征地农民基本生活保障制度。通过土地利用规划调整预留、宅基地置换等办法，增加农村集体非农业用地，发展二、三产业，增加农民投资性收入；组建农民经济合作组织，促进农村劳动力充分就业，增加农民工资性收入；大力兴办家庭农庄和服务基地，增加农民财产性收入。

　　3、全面启动一批重大工程。加快实施生活环境改善工程。投入1.6亿元全面解决农村饮水不安全问题。建立住房救助长效机制，解决农村贫困家庭的住房困难。新建沼气池1.2万口，同步改水改厕改圈1.2万户，发展“三沼”综合利用1.2万户。力争到2011年农村生活垃圾收集率达到90%，卫生厕所普及率达到85%。加快实施生产环境改善工程。四年投入4亿元整理国土40万亩，建设高产农田35万亩。完成县乡等级公路改造120公里，新建农村公路300公里，通达中心镇的公路达到二级或三级标准，通村和通达农业经济板块的公路基本达到四级标准。加快实施现代农业示范工程，以结构调整为主线推动现代农业发展。建设省级农业科技园5户，市级农业科技园30户，科技示范企业30家，科技示范基地30个。加快实施农民素质提升工程。加大劳动力就业培训，提高劳动者素质和技能，四年培训转移5万人。对乡镇综合文化站进行新一轮改造，实现村村建有农家书屋、篮球场及配套娱乐健身设施。

　　（三）围绕建设“两型社会”先行区，努力推进和谐发展

　　按照“两型社会”的要求重新规划和布局鄂州城乡发展体系，制定经济和社会发展战略。坚持走绿色、集约、高端化发展之路，促进经济社会发展与人口、资源、环境相协调，彰显鄂州的产业特色和生态优势，走出一条有别于传统模式的工业化、城市化发展新路，努力成为武汉城市圈“两型社会”建设的先行区和试验田。

　　1、强力推进新型工业化。坚定不移地走产业高端化之路，运用高新技术改造传统产业，加快装备大型化、现代化进程；鼓励企业对接高端产业链，在产品就地深、精加工上下工夫，以龙头企业带动上下游企业集聚，以优势产业吸引高端龙头企业加盟。加快培育有竞争力的支柱产业，力争钢铁产业销售收入达到500亿元，能源产业销售收入达到100亿元，电子信息、石化和建材产业销售收入分别达到50亿元，生物医药、新材料、纺织服装产业销售收入分别达到20亿元。通过实施重大项目提升技术层次、提高产品质量，力争到2011年省级名牌达30个以上，中国名牌2-3个，国家免检产品5-8个。积极支持企业上市，力争新增2-3个上市企业。大力发展循环经济，开展循环经济产业园区和重点产业项目试点工作。以鄂州开发区为依托，开展循环经济试点；抓好钢铁、能源、建材等重点行业、重点企业的节能减排工作；制定区域限批管理制度，严格禁止新建、扩建污染严重项目，完善高能耗、高污染企业退出机制，在城区划定的高污染燃料禁燃区和控制区内强制推广使用清洁能源。

　　2、强力推进新型城市化。以宜居宜业为目标，以“一主三新”为主体，努力构建功能互补、特色鲜明、城乡联动、绿色生态的组群式大城市。筹建凤凰大桥、双港大桥等5座桥梁，新建城市中轴线、希望大道等18条道路，完成主城区骨干路网刷黑工程，实施洋澜湖生态修复工程，建设雨台山公园、火车站站前广场等五个游园，建设科技馆、博物馆、艺术馆、体育馆等科技文化体育工程，建设沿江、长港等生态景观工程，建设“两区三城”污水处理厂和城区垃圾处理场。加大投入，改善老城区背街小巷环境。强力推进“四城联创”，努力提升城市层次和品位。将产业园区建设与城市新区建设结合起来，使产业园区成为实现城市扩张、功能提升的载体，为鄂州由中等城市向组群式大城市跨越提供支撑。将推动城镇化与农村新社区建设结合起来，投入资金1.8亿元加快特色镇基础设施建设，提高其对农民的吸纳能力、对产业的聚集能力和自身财政实力。将国土整理与迁村腾地和散居农户搬迁结合起来，使国土整理成为改善农业生产条件、改善农民生活环境的民心工程。将城市开发与生态保护结合起来，在城市规划和园区建设中推行环境评价，统筹兼顾经济效益、生态效益。认真做好城市品牌策划、推介工作，提高城市知名度和美誉度，进一步挖掘吴都文化，培育城市精神，提高城市凝聚力和向心力。

　　3、强力推进生活环境改善。高度重视生态文明建设，坚持把治理和保护统一起来，把工程措施与生态修复统一起来。出台《鄂州市山体保护规划》、《鄂州市湖泊保护规划》、《鄂州市地表水功能区划》，开展梁子湖、红莲湖、洋澜湖、花马湖等重点湖泊保护和综合治理。推进教育均衡发展，促进教育公平。完成农村学校布局调整和寄宿制学校改造，加强城乡教育设施建设，形成布局合理、体系健全、结构优化、适应需求的教育格局。大力发展职业教育，引进优势职教资源在鄂州办学，力争到2011年在校职校生达到5万人。健全市、区、乡镇（街道）、社区就业管理服务网络，加强职业技能培训，援助弱势群体就业。完善基本养老、失业、医疗、工伤、生育保险制度，探索建立多层次、广覆盖的医疗保险制度。建立完善城乡居民最低生活保障制度、城乡贫困居民医疗救助制度、教育救助制度、临时救助制度、住房救助制度、法律援助制度、灾害应急救助制度。加强农村疾病预防控制体系、妇幼保健体系和卫生监督体系建设，到2011年，基本形成覆盖城乡的公共卫生体系和医疗保障体系。继续稳定低生育水平，加大出生人口性别比综合治理力度。　依法管理民族宗教事务，规范宗教活动场所建设。大力倡导社会主义新风尚，引导市民自觉践行传统美德、社会公德和职业道德，开展多种形式的文化体育活动。

　　4、强力推进体制机制创新。推动新一轮思想大解放，营造敢闯善创、勇于试验的改革氛围。加快形成有利于创业的体制机制，建立健全以信息服务、技术服务、市场服务、资金服务、人才服务为主要内容的创业支持和服务体系。降低创业门槛，减少审批环节，缩短审批时限，凡是应该取消的行政审批项目坚决取消。　加快形成有利于促进科技与经济结合的体制机制，支持葛店高新区创业服务中心、红莲湖电子信息孵化基地、团结高新创业孵化基地和省金刚石生产力促进中心建设，提高科技产业的孵化能力。加快形成有利于资源节约和环境友好的体制机制，探索制定绿色GDP的考核体系，建立完善排污权交易制度、生态保护补偿机制和集约节约用地奖励政策。加快形成有利于降低成本、提高效能的体制机制，深化财政、投融资、流通、文化和行政管理体制改革，形成为民务实高效的政务环境。

　　三、2008年工作安排

　　2008年是全面贯彻党的十七大精神的第一年，是改革开放三十周年，也是新一届政府开局之年，做好今年的工作意义重大。全市经济社会发展的主要预期目标是：地区生产总值增长13.5%；财政总收入增长18%；　地方一般预算收入增长18%；全社会固定资产投资增长25%；社会消费品零售总额增长15%；外贸出口增长12%；招商引资总额增长35%；实际利用外资增长10%；城镇居民人均可支配收入增长11%；农民人均纯收入增长10%；城镇新增就业岗位2万个以上，城镇登记失业率控制在4.3%以内；人口自然增长率控制在5‰以内；万元GDP综合能耗下降4.6%；二氧化硫排放控制在3.66万吨以内，化学需氧量排放控制在1.42万吨以内。

　　上述目标是根据今后四年发展的总目标和当前鄂州发展的基础提出的，是指导性目标。主要经济指标均高于全省平均水平，是积极的，经过努力也是可以实现的。今年要重点抓好八个方面的工作：

　　（一）引进大项目，培育大产业。抓紧编制出台承接产业转移的总体规划，全力争取承接新一轮产业大转移。在深圳、东莞、南京、上海、杭州等地举行系列招商活动，精心筹备参加第三届“中博会”。争取引进亿元项目30个以上，力争引进一批5亿元和10亿元项目。集中力量加快重点项目建设，安排投资千万元以上项目160个，年度计划投资110亿元，其中工业投资项目80个，年度计划投资70亿元。继续实行项目领导责任制、季度调度制、目标考核制，及时解决项目实施过程中出现的问题，提高项目履约率、开工率、达产率。　支持鄂钢宽厚板、100万吨钢材深加工、50万吨无缝钢管等项目开工建设，争取鄂州球团厂二期项目开工，培育壮大冶金产业；积极做好电厂二期建设协调工作，力争年底建成试运行，做好三期工程的前期争取工作，培育壮大能源产业；抓住富士康、中芯国际落户武汉的机遇，积极发展配套产业，推动与信息产业厅共建信息产业园，与武汉大学共建地理空间信息产业基地，与华中科技共建激光加工基地，培育壮大信息产业；抓住80万吨乙烯开工建设的机遇，科学论证，做好PVC、武钢煤焦油深加工等项目的引进工作，培育壮大石化产业；力争世纪新峰二期等一批项目建成投产，培育壮大建材产业；进一步促进燕矶金刚石刀具、白浒镇铸造与泵业、梁子湖非金属矿业等特色产业发展壮大。

　　（二）启动一体化试点，推进新农村建设。作为全省城乡一体化试点城市，今年要在重点领域、关键环节起好步。高起点编制特色中心镇和中心村的发展规划，启动10个特色镇的基础设施建设。壮大水产、蔬菜、畜牧、林果等四大支柱产业，发展无公害绿色有机农产品，培植农产品品牌，力争新增无公害和绿色食品产地5个、产品标识5个。突破性发展农产品加工业，新增省级龙头企业3-5家、重点培育农产品加工企业20家。加快农村市场网络体系及科技信息网络建设，力争乡镇“万村千乡市场工程”覆盖率达到100%，村级达到50%。引导支持龙头企业、农民专业合作组织直接向城市超市、社区市场和便利店配送农产品，实现农超对接。巩固乡镇综合配套改革成果，完善“以钱养事”机制，进一步推进“乡财区管”。深入推进集体林权制度改革，启动农村社区股份合作社、土地股份合作社、专业合作经济组织改革试点，推进农村集体资产产权制度创新试点。继续加大扶贫开发力度，集中力量，整合资源，全面启动第二轮30个重点村整村推进扶贫开发工作。全面实施农村生态家园建设工程，深入开展“清洁乡村、美化家园”活动，推广高效益资源循环利用模式，改善农村生产生活环境。

　　（三）实施重点建设工程，增强园区带动能力。继续推动产业布局向沿江和高速公路沿线集中，项目向开发区园区集中，生产要素向优势产业集中。葛华科技新城重点抓好“四纵四横”道路建设并启动供水工程，全力创建国家级开发区；鄂州经济开发区重点抓好发展大道、薛家沟桥、新港路、青天湖路、华光路、月河南路等工程；红莲湖旅游新城重点抓好环湖道路、金碧大道、庙岭大道、香江大道等工程；花湖工贸新城重点抓好鄂城大道延伸工程，建设花（湖）杨（叶）大道等工程；梁子湖生态旅游区重点抓好梁子镇主街环境整治、梁子岛老街改造、游客广场建设等工程。启动汉鄂快速通道工程，推动武汉北湖化工新城、葛华科技新城、鄂州主城区、花湖工贸新城四位一体；启动红莲湖至东湖高新六路、六十至凤凰山对接工程，完成高新大道向东延伸工程，打通鄂州至武汉的8条通道，开辟梁子岛至武汉的水上通道，开通鄂州至武汉城际列车，实现与武汉的水路和铁路对接；加快华蒲路、葛湖路建设，实现市域内交通循环。

　　（四）建立引导约束机制，完成节能减排任务。加快制定和实施促进节能减排的市场准入标准、强制性能效标准和环保标准，尽快形成以政府为主导、企业为主体、全社会共同推进的格局。实施《鄂州市产业发展导向目录》，严格准入门槛，加快建立节能统计、监测、考核三大体系，实行环保节能一票否决。大力引进节能、节地、环保、科技型企业，加快先进节能、环保技术的推广应用，推进节能环保技改项目实施。支持鄂钢、电厂、球团厂等传统骨干企业进行设备更新和技术改造，重点实施鄂钢余热发电、电厂二期烟气脱硫、球团厂烟尘治理、工业用水循环再利用等工程。继续加大“七个专项”治理力度，依法淘汰技术落后、能耗较高的“五小企业”。建设主城区、葛华科技新城、红莲湖、梁子岛四座污水处理厂及其污水收集管网工程。降低煤炭在一次能源消费中的比例，推广生物能源、可再生能源，发展农村沼气，减少生活性二氧化硫排放。大力发展生态农业，控制农村面源污染。

　　（五）树立大城市理念，形成组群式格局。顺应建设组群式大城市的发展趋势，科学规划。聘请一流规划设计院编制完成《鄂州市城乡总体规划（2008-2020）》、“一主三新”控制性详规、城东港口经济区分区规划、黄柏山港口经济区分区规划、十个特色镇总体规划等规划任务。坚持大城市的标准，搞好建设。建立市区、主城和新城联动工作机制，统筹城区发展。支持各区依托开发区、产业园区加快新城建设和旧城改造步伐，进一步增强承载力和吸引力。投资10亿元，加快主城区建设，完善城市功能。启动城市中轴线工程，促进城东港口经济区、城西新区和主城建成区连成一体；借助汉鄂快速通道，建设城市外环线；续建寿昌大道、吴都大道、樊口大桥及接线道路，新建迎宾大道、洋澜南路等工程；抓好城区道路刷黑及基础设施维护，完成城市污水处理厂升级改造；加快小桥都市产业园建设，推动火车站客运站房改造；继续实施洋澜湖治理工程，推进沿江整治和滨江风光带工程建设；启动主城区防护林和牛头山综合开发工程；加快“城中村”改造。按照大城市的要求，强化管理。强力推进“四城联创”，对照国家验收标准找差距，抓建设，促管理，动员全市各阶层共同参与，力争取得明显成效。积极开展“楚天杯”创建活动。积极探索新的城市管理模式，推行城市管理重心下移，强化街办社区管理城市的责任，完善“两级管理、三级网络”体制，推进城市管理进社区、进乡镇，加强以社区为单位的小区综合治理和住宅区物业管理，进一步落实门前“四包”责任制，加大对违章建筑、占道经营的专项整治力度，探索和建立适应大城市发展的综合管理体制。

　　（六）改善发展环境，掀起创业热潮。出台支持全民创业的政策，鼓励机关事业单位工作人员带头创业，鼓励科技人员积极创业，鼓励外出经商务工人员回乡创业，鼓励农民进城创业，鼓励就业困难群体、大中专毕业生、军转干部、退役士兵、留学回归等群体自主创业。深入开展信用城市创建活动，引导各类商业银行、农村信用社加大对鄂州的产业支持力度和创业扶持力度。建立全民创业指导中心，为创业者提供创业培训、创业指导、项目开发等“一条龙”服务。加快武汉职业技术学院等职校的引进和建设步伐，扩大职业教育规模，为企业发展提供高技能人才和熟练工人保障。积极推进对外贸易，加强对外经济技术合作。出台《鄂州市服务业跨越发展规划纲要》，推动服务业跨越发展。加快引进大型物流集团，培育2家以上大型龙头物流企业。加快旅游业发展，大力引进大型旅游投资企业，鼓励建设高星级酒店；认真做好“中国优秀旅游城市”复核工作，积极筹办第四届华中旅游博览会参展活动和第九届梁子湖捕鱼旅游节。引导房地产业健康发展，新开工面积100万平方米。推动中介机构与行政事业单位脱钩，放开中介服务市场，鼓励和吸引更多的中介组织进入鄂州。继续推进中小企业成长工程，力争实现规模以上工业企业新增100家。

　　（七）高度重视民生，促进社会和谐。完成市八中建设，努力化解“普九”债务，完善农村义务教育经费保障机制，做好城区秋季义务教育阶段学生免除学杂费工作，做到学有所教。加大对企业用工的监督管理，加强对农民工的权益保护，加快建立企业职工工资正常增长和支付保障机制，做到劳有所得。逐步建立农村社会养老保险制度，提高农村新型合作医疗补助标准，扩大城镇职工基本医疗保险和城乡居民低保覆盖面，加快医疗基础设施建设，新增甲级村卫生室100家，新增2所惠民医院，做到病有所医。加快鄂州市福利中心建设，完善农村福利院基础设施，努力将愿意集中供养的五保对象全部纳入集中供养范围，做到老有所养。加快经济适用房建设步伐，确保建成7万平方米；完善廉租房制度，做好提标扩面工作；推进非公经济单位和农村中小学教师建立住房公积金制度，帮助农村特困家庭改造危房、新建住房，解决城乡低收入家庭住房困难，做到住有所居。加强价格监测、预警和监管，依法打击哄抬物价等各种价格违法违规行为，努力保持居民消费价格水平基本稳定。加大计划生育奖励政策的落实力度。加强应急管理，高度重视安全生产、食品药品安全、产品质量工作。加强社会治安综合治理，深入开展平安创建活动，依法防范和打击违法犯罪活动，保障人民群众安居乐业。规范网吧管理，加强公共文化服务体系建设，丰富群众精神文化生活。迎接北京奥运会，广泛开展全民健身活动。关心和支持老龄、青年、妇女、儿童事业发展，提高残疾人康复、教育、就业水平。开展第二次全国经济普查，摸准全市经济底数。做好民族宗教、外事侨务、广播电视、邮政、石油石化、烟草、粮食、供电、金融、保险、通讯、防震减灾、档案、方志、人防、消防、农机、气象等工作。

　　（八）办好“十件实事”，解决“三最”问题。围绕解决群众最关心、最直接、最现实的利益问题，努力办好“十件实事”：1、改善农村生产生活条件。筹资4000万元，使11个乡镇43个村10万人喝上自来水；筹资1亿元，实施7万亩国土整理项目；筹资2600万元，实施排灌泵站更新改造工程；筹资1200万元，完成300个自然湾塘堰的清淤改造。2、改善城镇居民生产生活环境。筹资2000万元，改造老城区背街小巷16条，硬化道路3万平方米，完成排水、环卫、园林、路灯、路牌、门牌等配套工程；筹资6000万元，改善十个特色镇基础设施。3、优先发展城乡公交。筹资41万元，建设农村客运候车棚20座、招呼站58处；筹资810万元，在城东、城南、城西各建设公交站场一处；延伸5条公交线路到农村，更新城市公交站牌、公交车标志牌。4、改善农村办学条件。筹资500万元，对5所农村学校实施“三建两改”工程。5、加强社区场所和卫生设施建设。　　筹资320万元，新、改、扩建8个标准化（300平方米以上）社区活动场所；筹资420万元，新、改建3个卫生服务中心、4个社区卫生服务站，为居民提供公共卫生服务。6、解决城乡特困家庭住房困难。筹资480万元，全部解决570户农村特困家庭住房困难；筹资800万元，解决200户城镇低收入人群住房困难；筹资150万元，对1500户城镇特困住房户实行住房租赁补贴和租金核减。7、多渠道扩大就业。增加500万元小额担保基金规模，扶持下岗失业人员再就业；筹资700万元，培训转移劳动力20000人；筹资600万元，扶持100个创业带头人，带动1000名下岗失业人员再就业。8、提高市民健康保障能力。筹资2400万元，启动城镇居民医疗保险试点工作；扩大新生儿免疫规划。9、筹资650万元，推行“煤改气”工程和油烟噪声整治工程。在城区扩大1万户居民使用天然气。10、加大文化活动场所建设力度。筹资300万元，在150个村建设农家书屋；筹资5000万元，启动吴都博物馆建设，改善湖北省京剧二团条件，改建鄂州市艺术馆。

　　四、加强政府自身建设

　　新的形势、新的任务，对政府工作提出了新的要求。新一届政府将继续秉持“用心做事、公正待人、发展为先、人民至上”的理念，全面提高组织和领导发展的能力，建设一个对人民负责、让人民满意的政府。

　　一是切实提高科学发展的能力。加强学习型机关建设，提高推动科学发展的能力和水平。对发展机遇保持高度紧迫，进一步解放思想，更新观念，始终保持与时俱进、奋发向上的蓬勃朝气，以更大的胆识、更大的魄力、更大的力度，推动鄂州经济跨越式发展；对发展形势保持高度关注，学会用全球视野谋划工作、长远意识处理事务、全局观念解决矛盾；对发展目标保持高远追求，以民不富心不安的事业心，以工作无为终身有愧的进取心，奋勇争先，不懈努力，兢兢业业，精益求精；对发展中的困难保持清醒认识，不计名利，不避嫌怨，直面应对，攻坚克难。要把“两个率先”作为全市人民共同奋斗的目标，把节能减排、保护环境作为落实科学发展观、转变发展方式的突破口和重要抓手，把建设宜居宜业大城市作为矢志不渝、努力前进的方向，让鄂州的实力更强、市民更富、城市更美。

　　二是切实提高促进和谐的能力。社会和谐稳定，百姓安居乐业，是全市人民的共同愿望，也是政府工作的首要任务。要把构建“和谐鄂州”摆到突出位置，努力形成全体人民各尽其能、各得其所而又和谐相处的社会局面。从市民最关心的现实问题入手，倾听民声、反映民意、集中民智、体现民利，努力破解劳动就业、社会保障、医疗卫生、食品安全、教育收费、安全生产、环境污染等问题。

　　认真做好信访工作，引导群众以理性合法的形式表达利益诉求。对可能影响和谐稳定的问题，要见事早、行动快、解决好，力保一方平安。加快完善各项应急预案，提高政府应对各种灾害和突发事件的能力，做到既善于在常态下进行管理，又善于在非常态下进行管理。

　　三是切实提高服务市民的能力。创新政府管理理念、管理体制机制、管理方式和管理手段，寓管理于服务之中，提升公务员素质，更好地为基层、企业和社会公众服务。将高效便民作为政府制定工作流程的第一原则，推行网上审批、简化程序、限时办结，做到公开、公正、公平，方便群众办事。将畅通市民诉求渠道作为政府服务市民的第一抓手，完善社情民意反映制度、重大事项公示制度、社会听证制度和政府信息公开制度，通过“亲民热线”、“市长在线访谈”、“市长专线”、“市长信箱”等沟通平台加强政府与市民的互动，让广大市民有机会说话、有地方讲理、有人帮助解决问题。

四是切实提高依法行政的能力。积极推进法治政府和法制社会建设。牢固树立依法行政理念，把依法行政贯穿于政府决策、执行、监督的全过程。建立完善公众参与、专家论证和政府决策相结合的行政决策机制，进一步落实行政决策程序和规则。严格规范行政执法行为，认真做好行政复议和行政应诉工作，推行行政首长出庭应诉制度。坚持市政府与市人大、市政协联系会议制度。认真办理人大代表建议和政协委员提案。主动向人大报告工作，自觉接受人大及其常委会的法律监督和工作监督。自觉接受政协的民主监督，主动听取各民主党派、工商联、无党派人士和人民团体的意见。自觉接受新闻舆论和社会公众监督。建设法制社会，政府和市民均有依法行事的责任。加强法律知识的普及和法律意识的培养，引导市民以法律为准绳处理各种矛盾纠纷。

　　五是切实提高廉洁从政的能力。全面落实党风廉政建设责任制，加强反腐倡廉制度建设，形成靠制度管人制权理事的有效机制。着力规范市场交易行为，认真落实工程招投标、产权交易、土地出让、政府采购等工作制度，深入开展治理商业贿赂专项工作，坚决纠正损害群众利益的不正之风。坚持艰苦奋斗、勤俭节约，反对铺张浪费、奢靡之风。规范部门财务收支行为，支持监察、审计等部门依法履行监督职责。大力加强政风建设。不断强化目标管理责任制和行政问责制，健全抓落实的工作机制，加强政务督察、限时督办和效能监察，确保政令畅通和各项工作落到实处。对政府工作人员严格要求、严格教育、严格管理、严格监督，大兴敢抓敢管、勇于创新、勤政廉洁、团结协作之风，以良好形象赢得人民群众的信任和支持。

　　各位代表，鄂州改革开放和现代化建设正在进入一个新的发展阶段。在新的起点上加快实现鄂州的振兴崛起，是时代赋予我们的光荣使命，是全市人民对我们的殷切期待，是我们必须履行的神圣职责。让我们紧密团结在以胡锦涛同志为总书记的党中央周围，高举中国特色社会主义伟大旗帜，在市委的坚强领导下，紧紧依靠全市人民，同心同德，锐意进取，励精图治，真抓实干，为开创鄂州更加美好的未来而努力奋斗！
